# *catena*-Poly[[bis­(pyridine-κ*N*)zinc]-μ-5-carb­oxy­benzene-1,3-di­carboxyl­ato-κ^2^ *O* ^1^:*O* ^3^]

**DOI:** 10.1107/S1600536813014347

**Published:** 2013-05-31

**Authors:** Bunlawee Yotnoi, Apinpus Rujiwatra

**Affiliations:** a Division of Chemistry, School of Science, University of Phayao, Phayao 56000, Thailand; b Department of Chemistry, Faculty of Science, Chiang Mai University, Chiang Mai 50200, Thailand

## Abstract

The title one-dimensional coordination polymer, [Zn(C_9_H_4_O_6_)(C_5_H_5_N)_2_]_*n*_ or [Zn(HBTC)(py)_2_]_*n*_, (I), where BTC is benzene-1,3,5-tricarboxylate and py is pyridine, is a solvent-free polymorph of [Zn(HBTC)(py)_2_]·2C_2_H_5_OH [Yaghi *et al.* (1997 ▶). *Chem. Mater.* **9**, 1074–1076]. Differences in the spatial arrangements and supra­molecular packing of the [Zn(HBTC)(py)_2_]_*n*_ chains in the two structures are described. The chain in (I) extends parallel to [100] and is severely puckered, with a Zn⋯Zn distance of 8.3599 (3) Å and a Zn⋯Zn⋯Zn angle of 107.516 (3)°, as a result of hydrogen-bonding inter­actions of the types O—H⋯O and C—H⋯O. There is no evidence for π–π inter­actions in (I). The differences between the solvent-free and solvent-containing structures can be accounted for by the absence of the ethanol solvent mol­ecule and the use of the converging pair of O atoms in the bis-monodentate bridging HBTC^2−^ ligand in (I).

## Related literature

For the ethanol monosolvate of (I), see: Yaghi *et al.* (1997 ▶). For a review on supra­molecular isomerism in coordination compounds, see: Zhang *et al.* (2009 ▶). 
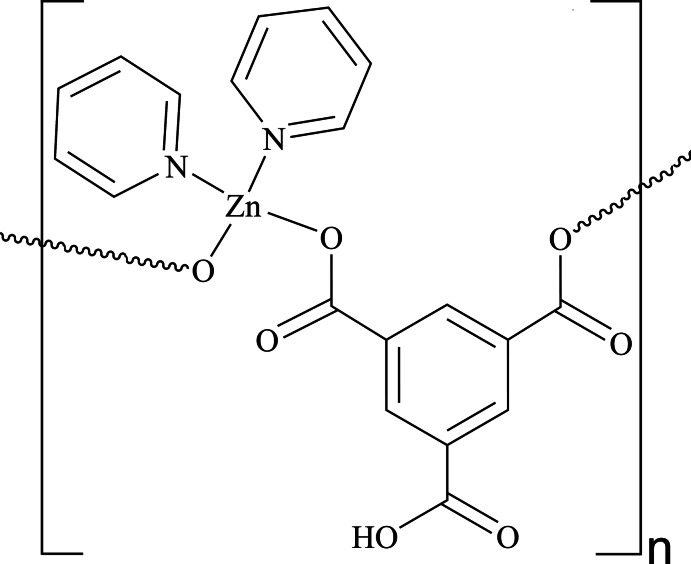


## Experimental

### 

#### Crystal data

[Zn(C_9_H_4_O_6_)(C_5_H_5_N)_2_]
*M* *_r_* = 431.69
Orthorhombic, 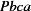

*a* = 13.4850 (4) Å
*b* = 15.7677 (4) Å
*c* = 16.7252 (4) Å
*V* = 3556.24 (16) Å^3^
*Z* = 8
Mo *K*α radiation
μ = 1.42 mm^−1^
*T* = 293 K
0.40 × 0.32 × 0.20 mm

#### Data collection

Bruker SMART CCD area-detector diffractometer
Absorption correction: multi-scan (*SADABS*; Bruker, 2001 ▶) *T*_min_ = 0.638, *T*_max_ = 0.746
18851 measured reflections
4402 independent reflections
3448 reflections with *I* > 2σ(*I*)
*R*_int_ = 0.027

#### Refinement

*R*[*F* ^2^ > 2σ(*F* ^2^)] = 0.028
*wR*(*F* ^2^) = 0.075
*S* = 1.03
4402 reflections
257 parameters
H atoms treated by a mixture of independent and constrained refinement
Δρ_max_ = 0.32 e Å^−3^
Δρ_min_ = −0.27 e Å^−3^

### 

Data collection: *APEX2* (Bruker, 2007 ▶); cell refinement: *SAINT* (Bruker, 2007 ▶); data reduction: *SAINT*; program(s) used to solve structure: *SHELXS97* (Sheldrick, 2008 ▶) and *WinGX* (Farrugia, 2012 ▶); program(s) used to refine structure: *SHELXL2013* (Sheldrick, 2008 ▶) and *WinGX*; molecular graphics: *DIAMOND* (Brandenburg, 1999 ▶); software used to prepare material for publication: *publCIF* (Westrip, 2010 ▶).

## Supplementary Material

Crystal structure: contains datablock(s) I, global. DOI: 10.1107/S1600536813014347/cq2004sup1.cif

Structure factors: contains datablock(s) I. DOI: 10.1107/S1600536813014347/cq2004Isup2.hkl

Additional supplementary materials:  crystallographic information; 3D view; checkCIF report

## Figures and Tables

**Table 1:** Hydrogen-bond geometry (Å, °)

*D*—H⋯*A*	*D*—H	H⋯*A*	*D*⋯*A*	*D*—H⋯*A*
O5—H5*W*⋯O3^i^	0.86 (3)	1.74 (3)	2.5813 (19)	164 (3)
C1—H1⋯O1	0.93	2.45	3.053 (2)	122
C5—H5⋯O6^ii^	0.93	2.39	3.129 (3)	136
C17—H17⋯O5	0.93	2.37	2.693 (2)	100
C17—H17⋯O5^i^	0.93	2.42	3.309 (2)	159

Symmetry codes: (i) 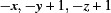; (ii) 
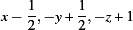
.

**Fig. 1.**
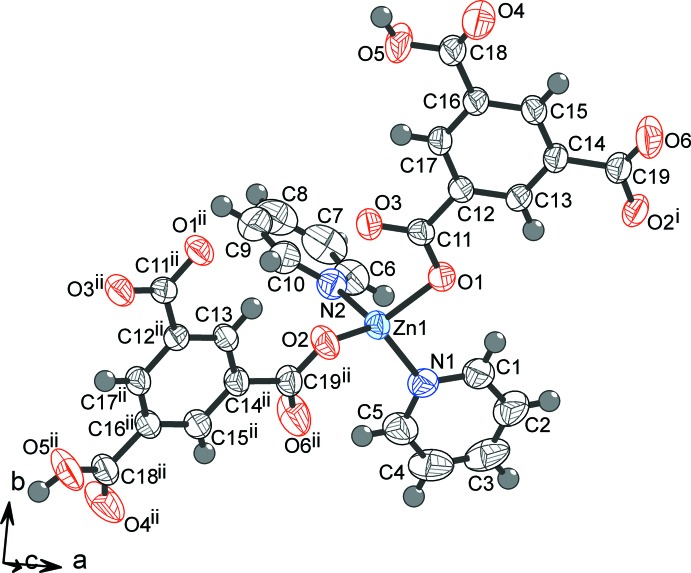
View of an extended unit of I with atom numbering scheme. Displacement ellipsoids are drawn at the 70% probability level. [Symmetry codes: (i) 1/2 + x, 0.5 - y, 1 - z; (ii) -1/2 + x, 0.5 - y, 1 - z]

**Fig. 2.**
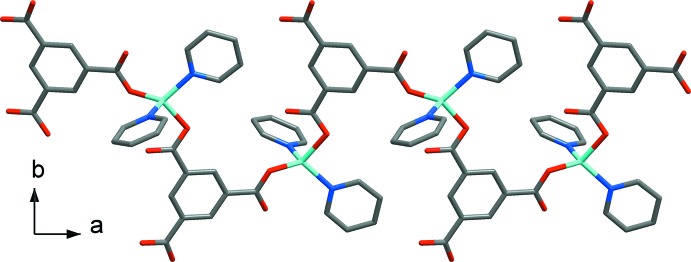
View of the one-dimensional chain of I. Hydrogen atoms are omitted for clarity.

**Fig. 3.**
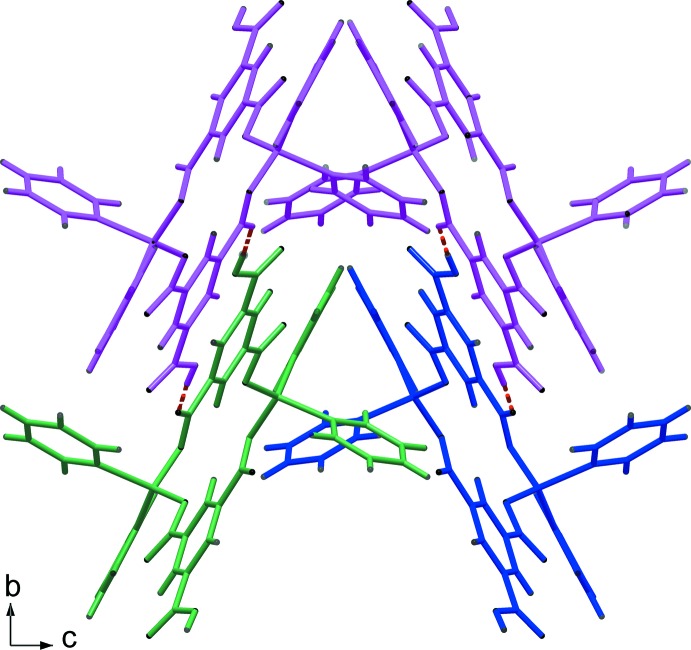
View of supramolecular packing in I, directed primarily by the O—H···O hydrogen bonding interactions (dotted lines).

## supplementary crystallographic information

### Comment

The reported complex [Zn(HBTC)(py)_2_]*_n_* (I), where BTC = 1,3,5-benzenetricarboxylate and pyr = pyridine (C_5_H_5_N), is a supramolecular isomer of the previously reported [Zn(HBTC)(py)_2_].2C_2_H_5_OH (Yaghi *et al.*, 1997). It is clear that the two isomers result from differences in the synthesis method. In order to synthesize I, a simple solution route was adopted, whereas the crystals of [Zn(HBTC)(py)_2_].2C_2_H_5_OH have only been synthesized when poly(ethyleneoxide) (PEO) gel is used as the reaction medium in the presence of C_2_H_5_OH as solvent (Yaghi *et al.*, 1997). The absence or presence of the C_2_H_5_OH solvent molecules results in different spatial arrangements and supramolecular packing in the two isomers.

While the two supramolecular isomers are built up of the same structural building motifs, *i.e.* a tetrahedral Zn(II) ion, two monodentate pyr molecules and a bridging HBTC^2-^ anion, the architecture of the one-dimensional chains of the two isomers notably differs. In the case of I, an asymmetric unit comprises a tetrahedral Zn(II) ion (Fig. 1), coordinated by two N atoms (N1 and N2) from two crystallographically independent pyr molecules and two O atoms (O1 and O2) from two distinct carboxylato groups of the HBTC^2-^ anion. The HBTC^2-^ ligand in structure I adopts a *bis*-monodentate bridging mode (µ_2_-η^1^:η^1^:η^0^:η^0^:η^0^:η^0^) (Fig. 2), linking two adjacent Zn(II) ions to form a one-dimensional chain of chemical composition [Zn(HBTC)(py)_2_]*_n_*, extending in the [1 0 0] direction. Because of the converging positions of the coordinating O1 and O2 atoms, the derived chain is severely puckered with a Zn···Zn distance of 8.3599 (3) Å and a Zn···Zn···Zn angle of 107.516 (3)°. In comparison, the one-dimensional chain of the previously reported [Zn(HBTC)(py)_2_].2C_2_H_5_OH isomer is almost linear with corresponding distance and angle values of 10.162 (2) Å and 180°, respectively. The presence of the C_2_H_5_OH solvent molecule in the vicinity of the two coordinating carboxylato groups in the [Zn(HBTC)(py)_2_].2C_2_H_5_OH isomer ostensibly forces the coordinating atoms to be the diverging pair of O atoms (equivalent to O3 and O6 in I), rather than the converging pair as in I.

A larger degree of puckering in I compared to that of the previously reported isomer imparts an immense influence on the corresponding supramolecular interactions and packing. While there are no π–π interactions in I, which is in contrast to the [Zn(HBTC)(py)_2_].2C_2_H_5_OH case, an acidic proton of the bridging HBTC^2-^ anion is involved in the hydrogen bonding interactions with the O atom of the HBTC^2-^ anion from the adjacent chain (Fig. 3). Weak C—H···O hydrogen bonding interactions further link these one-dimensional chains into a dense, three-dimensional supramolecular structure.

### Experimental

A small fraction of pyridine (0.50 ml, 6.18 mmol; 99.8% Sigma-Aldrich) was gradually added to a 2.50 ml of a 0.204 molL^-1^ aqueous solution of 1,3,5-benzenetricarboxylic acid (H_3_BTC; 0.509 mmol; 95% Sigma-Aldrich) with stirring until the acid was completely dissolved. Then 2.50 ml of 0.296 molL^-1^ ZnSO_4_.7H_2_O (aq) solution (0.741 mmol; 99% Sigma-Aldrich) was added, giving a clear solution. The solution was left at ambient temperature for 2 days before the colourless crystals of I started to crystallize.

### Refinement

Atom H5W atom was located in a difference Fourier map. The aromatic H-atoms were treated as riding groups on the bonded C-atoms using a C—H bond length of 0.93 Å.

### Crystal data

**Table:**

[Zn(C_9_H_4_O_6_)(C_5_H_5_N)_2_]	*D*_x_ = 1.613 Mg m^−^^3^
*M**_r_* = 431.69	Mo *K*α radiation, λ = 0.71073 Å
Orthorhombic, *P**b**c**a*	Cell parameters from 12396 reflections
*a* = 13.4850 (4) Å	θ = 2.3–28.3°
*b* = 15.7677 (4) Å	µ = 1.42 mm^−^^1^
*c* = 16.7252 (4) Å	*T* = 293 K
*V* = 3556.24 (16) Å^3^	Block, colorless
*Z* = 8	0.40 × 0.32 × 0.20 mm
*F*(000) = 1760	

### Data collection

**Table:**

Bruker SMART CCD area-detector diffractometer	3448 reflections with *I* > 2σ(*I*)
Radiation source: fine-focus sealed tube	*R*_int_ = 0.027
ω scan	θ_max_ = 28.3°, θ_min_ = 2.3°
Absorption correction: multi-scan (*SADABS*; Bruker, 2001)	*h* = −17→16
*T*_min_ = 0.638, *T*_max_ = 0.746	*k* = −18→21
18851 measured reflections	*l* = −22→18
4402 independent reflections	

### Refinement

**Table:**

Refinement on *F*^2^	0 restraints
Least-squares matrix: full	Hydrogen site location: mixed
*R*[*F*^2^ > 2σ(*F*^2^)] = 0.028	H atoms treated by a mixture of independent and constrained refinement
*wR*(*F*^2^) = 0.075	*w* = 1/[σ^2^(*F*_o_^2^) + (0.0343*P*)^2^ + 1.2882*P*] where *P* = (*F*_o_^2^ + 2*F*_c_^2^)/3
*S* = 1.03	(Δ/σ)_max_ = 0.001
4402 reflections	Δρ_max_ = 0.32 e Å^−^^3^
257 parameters	Δρ_min_ = −0.27 e Å^−^^3^

### Special details

**Table:**

Geometry. All e.s.d.'s (except the e.s.d. in the dihedral angle between two l.s. planes) are estimated using the full covariance matrix. The cell e.s.d.'s are taken into account individually in the estimation of e.s.d.'s in distances, angles and torsion angles; correlations between e.s.d.'s in cell parameters are only used when they are defined by crystal symmetry. An approximate (isotropic) treatment of cell e.s.d.'s is used for estimating e.s.d.'s involving l.s. planes.

### Fractional atomic coordinates and isotropic or equivalent isotropic displacement parameters (Å^2^)

**Table:**

	*x*	*y*	*z*	*U*_iso_*/*U*_eq_	
Zn1	0.13687 (2)	0.15689 (2)	0.38115 (2)	0.02934 (7)	
O1	0.22574 (9)	0.24099 (8)	0.43444 (8)	0.0387 (3)	
O2	0.02076 (9)	0.12804 (8)	0.44224 (8)	0.0380 (3)	
O3	0.08532 (9)	0.31056 (8)	0.42911 (9)	0.0403 (3)	
O4	0.17223 (13)	0.64303 (10)	0.62643 (11)	0.0636 (5)	
O5	0.06430 (11)	0.59328 (10)	0.53854 (11)	0.0610 (5)	
O6	0.50499 (11)	0.48839 (11)	0.63064 (10)	0.0572 (4)	
N1	0.24456 (11)	0.07341 (9)	0.34795 (9)	0.0344 (3)	
N2	0.08742 (12)	0.20600 (9)	0.27386 (9)	0.0351 (3)	
C1	0.34018 (14)	0.09488 (13)	0.35799 (13)	0.0403 (4)	
H1	0.3551	0.1456	0.3837	0.048*	
C2	0.41665 (16)	0.04472 (14)	0.33178 (15)	0.0526 (6)	
H2	0.4821	0.0612	0.3398	0.063*	
C3	0.39544 (18)	−0.02992 (14)	0.29374 (14)	0.0554 (6)	
H3	0.4463	−0.0645	0.2749	0.066*	
C4	0.29877 (19)	−0.05299 (13)	0.28377 (14)	0.0554 (6)	
H4	0.2829	−0.1038	0.2586	0.066*	
C5	0.22515 (16)	−0.00031 (13)	0.31132 (12)	0.0451 (5)	
H5	0.1594	−0.0164	0.3043	0.054*	
C6	0.14604 (16)	0.21440 (13)	0.21013 (12)	0.0455 (5)	
H6	0.2091	0.1906	0.2119	0.055*	
C7	0.1175 (2)	0.25651 (16)	0.14217 (13)	0.0581 (6)	
H7	0.1607	0.2614	0.0991	0.070*	
C8	0.0245 (2)	0.29120 (16)	0.13863 (15)	0.0639 (7)	
H8	0.0034	0.3200	0.0932	0.077*	
C9	−0.03721 (19)	0.28258 (14)	0.20360 (15)	0.0570 (6)	
H9	−0.1010	0.3050	0.2025	0.068*	
C10	−0.00334 (15)	0.24024 (12)	0.27043 (12)	0.0424 (5)	
H10	−0.0449	0.2353	0.3145	0.051*	
C11	0.17367 (13)	0.30629 (11)	0.44909 (10)	0.0290 (3)	
C12	0.22248 (12)	0.37837 (10)	0.49276 (10)	0.0274 (3)	
C13	0.32159 (13)	0.37427 (11)	0.51554 (10)	0.0290 (4)	
H13	0.3589	0.3265	0.5029	0.035*	
C14	0.36539 (12)	0.44093 (11)	0.55706 (10)	0.0296 (4)	
C15	0.30929 (13)	0.51149 (11)	0.57672 (11)	0.0325 (4)	
H15	0.3381	0.5560	0.6048	0.039*	
C16	0.20993 (12)	0.51606 (11)	0.55457 (11)	0.0311 (4)	
C17	0.16744 (13)	0.44966 (11)	0.51237 (11)	0.0306 (4)	
H17	0.1013	0.4530	0.4970	0.037*	
C18	0.14864 (13)	0.59105 (12)	0.57802 (13)	0.0377 (4)	
C19	0.47177 (13)	0.43569 (11)	0.58439 (11)	0.0322 (4)	
H5W	0.022 (2)	0.6310 (19)	0.5540 (18)	0.096 (10)*	

### Atomic displacement parameters (Å^2^)

**Table:**

	*U*^11^	*U*^22^	*U*^33^	*U*^12^	*U*^13^	*U*^23^
Zn1	0.02247 (11)	0.02948 (11)	0.03608 (12)	−0.00104 (8)	0.00170 (8)	−0.00546 (8)
O1	0.0273 (7)	0.0301 (6)	0.0587 (8)	0.0041 (5)	−0.0047 (6)	−0.0160 (6)
O2	0.0269 (7)	0.0399 (7)	0.0472 (7)	−0.0100 (5)	0.0077 (6)	−0.0080 (6)
O3	0.0262 (7)	0.0345 (7)	0.0604 (9)	0.0031 (5)	−0.0077 (6)	−0.0175 (6)
O4	0.0489 (9)	0.0533 (10)	0.0887 (13)	0.0178 (8)	−0.0231 (9)	−0.0421 (9)
O5	0.0303 (8)	0.0560 (10)	0.0967 (13)	0.0175 (7)	−0.0177 (8)	−0.0409 (9)
O6	0.0325 (8)	0.0634 (10)	0.0759 (11)	0.0070 (7)	−0.0159 (7)	−0.0354 (8)
N1	0.0317 (8)	0.0309 (8)	0.0407 (8)	0.0036 (6)	0.0055 (7)	−0.0024 (7)
N2	0.0352 (9)	0.0350 (8)	0.0352 (8)	0.0005 (7)	−0.0019 (7)	−0.0053 (7)
C1	0.0338 (10)	0.0344 (10)	0.0526 (11)	0.0012 (8)	0.0058 (9)	−0.0005 (9)
C2	0.0342 (11)	0.0496 (12)	0.0740 (16)	0.0100 (9)	0.0150 (10)	0.0077 (11)
C3	0.0593 (15)	0.0445 (12)	0.0625 (14)	0.0220 (11)	0.0265 (12)	0.0054 (11)
C4	0.0711 (16)	0.0364 (11)	0.0587 (14)	0.0090 (11)	0.0144 (12)	−0.0115 (10)
C5	0.0442 (12)	0.0368 (10)	0.0544 (12)	0.0002 (9)	0.0051 (10)	−0.0112 (9)
C6	0.0488 (13)	0.0469 (11)	0.0408 (11)	−0.0019 (10)	0.0059 (9)	−0.0068 (9)
C7	0.0817 (18)	0.0554 (14)	0.0373 (11)	−0.0166 (13)	0.0046 (11)	−0.0009 (11)
C8	0.094 (2)	0.0476 (13)	0.0500 (14)	−0.0149 (14)	−0.0259 (14)	0.0094 (11)
C9	0.0558 (15)	0.0443 (12)	0.0710 (16)	0.0029 (11)	−0.0223 (12)	0.0006 (11)
C10	0.0403 (11)	0.0397 (10)	0.0472 (11)	0.0016 (9)	−0.0036 (9)	−0.0043 (9)
C11	0.0273 (8)	0.0256 (8)	0.0342 (9)	−0.0003 (7)	0.0012 (7)	−0.0049 (7)
C12	0.0239 (8)	0.0256 (8)	0.0328 (8)	−0.0012 (6)	0.0005 (7)	−0.0037 (7)
C13	0.0239 (8)	0.0272 (8)	0.0358 (9)	0.0041 (7)	0.0006 (7)	−0.0042 (7)
C14	0.0224 (8)	0.0321 (9)	0.0342 (9)	0.0018 (7)	−0.0015 (7)	−0.0028 (7)
C15	0.0258 (9)	0.0304 (9)	0.0414 (10)	−0.0007 (7)	−0.0038 (7)	−0.0100 (8)
C16	0.0242 (8)	0.0287 (8)	0.0404 (9)	0.0023 (7)	−0.0015 (7)	−0.0080 (7)
C17	0.0215 (8)	0.0300 (9)	0.0404 (10)	0.0008 (7)	−0.0027 (7)	−0.0057 (7)
C18	0.0256 (9)	0.0338 (9)	0.0536 (12)	0.0040 (7)	−0.0019 (8)	−0.0114 (9)
C19	0.0246 (8)	0.0359 (9)	0.0361 (9)	0.0021 (7)	−0.0013 (7)	−0.0018 (8)

### Geometric parameters (Å, º)

**Table:**

Zn1—O2	1.9241 (12)	C5—H5	0.9300
Zn1—O1	1.9973 (12)	C6—C7	1.371 (3)
Zn1—N1	2.0371 (14)	C6—H6	0.9300
Zn1—N2	2.0650 (15)	C7—C8	1.370 (4)
O1—C11	1.270 (2)	C7—H7	0.9300
O2—C19^i^	1.283 (2)	C8—C9	1.375 (4)
O3—C11	1.239 (2)	C8—H8	0.9300
O4—C18	1.195 (2)	C9—C10	1.380 (3)
O5—C18	1.315 (2)	C9—H9	0.9300
O5—H5W	0.86 (3)	C10—H10	0.9300
O6—C19	1.220 (2)	C11—C12	1.503 (2)
N1—C5	1.340 (2)	C12—C17	1.386 (2)
N1—C1	1.344 (2)	C12—C13	1.391 (2)
N2—C6	1.334 (3)	C13—C14	1.391 (2)
N2—C10	1.339 (2)	C13—H13	0.9300
C1—C2	1.372 (3)	C14—C15	1.385 (2)
C1—H1	0.9300	C14—C19	1.508 (2)
C2—C3	1.368 (3)	C15—C16	1.392 (2)
C2—H2	0.9300	C15—H15	0.9300
C3—C4	1.364 (3)	C16—C17	1.387 (2)
C3—H3	0.9300	C16—C18	1.495 (2)
C4—C5	1.374 (3)	C17—H17	0.9300
C4—H4	0.9300	C19—O2^ii^	1.283 (2)
			
O2—Zn1—O1	114.10 (5)	C7—C8—C9	118.7 (2)
O2—Zn1—N1	124.90 (6)	C7—C8—H8	120.6
O1—Zn1—N1	97.06 (6)	C9—C8—H8	120.6
O2—Zn1—N2	106.70 (6)	C8—C9—C10	119.2 (2)
O1—Zn1—N2	109.43 (6)	C8—C9—H9	120.4
N1—Zn1—N2	103.63 (6)	C10—C9—H9	120.4
C11—O1—Zn1	107.01 (11)	N2—C10—C9	122.2 (2)
C19^i^—O2—Zn1	114.84 (11)	N2—C10—H10	118.9
C18—O5—H5W	116 (2)	C9—C10—H10	118.9
C5—N1—C1	117.60 (17)	O3—C11—O1	121.57 (15)
C5—N1—Zn1	123.09 (13)	O3—C11—C12	120.73 (15)
C1—N1—Zn1	119.16 (13)	O1—C11—C12	117.69 (15)
C6—N2—C10	117.87 (18)	C17—C12—C13	119.16 (15)
C6—N2—Zn1	122.71 (14)	C17—C12—C11	119.58 (15)
C10—N2—Zn1	118.92 (13)	C13—C12—C11	121.25 (15)
N1—C1—C2	122.4 (2)	C12—C13—C14	120.63 (15)
N1—C1—H1	118.8	C12—C13—H13	119.7
C2—C1—H1	118.8	C14—C13—H13	119.7
C3—C2—C1	119.2 (2)	C15—C14—C13	119.57 (15)
C3—C2—H2	120.4	C15—C14—C19	119.45 (15)
C1—C2—H2	120.4	C13—C14—C19	120.92 (15)
C4—C3—C2	119.09 (19)	C14—C15—C16	120.28 (16)
C4—C3—H3	120.5	C14—C15—H15	119.9
C2—C3—H3	120.5	C16—C15—H15	119.9
C3—C4—C5	119.2 (2)	C17—C16—C15	119.61 (15)
C3—C4—H4	120.4	C17—C16—C18	120.15 (15)
C5—C4—H4	120.4	C15—C16—C18	120.22 (15)
N1—C5—C4	122.5 (2)	C12—C17—C16	120.75 (16)
N1—C5—H5	118.8	C12—C17—H17	119.6
C4—C5—H5	118.8	C16—C17—H17	119.6
N2—C6—C7	123.0 (2)	O4—C18—O5	123.49 (18)
N2—C6—H6	118.5	O4—C18—C16	124.95 (17)
C7—C6—H6	118.5	O5—C18—C16	111.55 (16)
C8—C7—C6	119.1 (2)	O6—C19—O2^ii^	124.36 (17)
C8—C7—H7	120.5	O6—C19—C14	120.27 (16)
C6—C7—H7	120.5	O2^ii^—C19—C14	115.32 (15)

Symmetry codes: (i) *x*−1/2, −*y*+1/2, −*z*+1; (ii) *x*+1/2, −*y*+1/2, −*z*+1.

### Hydrogen-bond geometry (Å, º)

**Table:**

*D*—H···*A*	*D*—H	H···*A*	*D*···*A*	*D*—H···*A*
O5—H5*W*···O3^iii^	0.86 (3)	1.74 (3)	2.5813 (19)	164 (3)
C1—H1···O1	0.93	2.45	3.053 (2)	122
C5—H5···O6^i^	0.93	2.39	3.129 (3)	136
C17—H17···O5	0.93	2.37	2.693 (2)	100
C17—H17···O5^iii^	0.93	2.42	3.309 (2)	159

Symmetry codes: (i) *x*−1/2, −*y*+1/2, −*z*+1; (iii) −*x*, −*y*+1, −*z*+1.
